# A network model of genomic hormone interactions underlying dementia and its translational validation through serendipitous off-target effect

**DOI:** 10.1186/1479-5876-11-177

**Published:** 2013-07-26

**Authors:** Erfan Younesi, Martin Hofmann-Apitius

**Affiliations:** 1 Department of Bioinformatics, Fraunhofer Institute for Algorithms and Scientific Computing (SCAI), Schloss Birlinghoven, Sankt Augustin, 53754, Germany; 2 Bonn-Aachen International Center for Information Technology, University of Bonn, Bonn, Germany

**Keywords:** Hormone, Dementia, Network model, Translational validation, Off-target effect

## Abstract

**Background:**

While the majority of studies have focused on the association between sex hormones and dementia, emerging evidence supports the role of other hormone signals in increasing dementia risk. However, due to the lack of an integrated view on mechanistic interactions of hormone signaling pathways associated with dementia, molecular mechanisms through which hormones contribute to the increased risk of dementia has remained unclear and capacity of translating hormone signals to potential therapeutic and diagnostic applications in relation to dementia has been undervalued.

**Methods:**

Using an integrative knowledge- and data-driven approach, a global hormone interaction network in the context of dementia was constructed, which was further filtered down to a model of convergent hormone signaling pathways. This model was evaluated for its biological and clinical relevance through pathway recovery test, evidence-based analysis, and biomarker-guided analysis. Translational validation of the model was performed using the proposed novel mechanism discovery approach based on ‘serendipitous off-target effects’.

**Results:**

Our results reveal the existence of a well-connected hormone interaction network underlying dementia. Seven hormone signaling pathways converge at the core of the hormone interaction network, which are shown to be mechanistically linked to the risk of dementia. Amongst these pathways, estrogen signaling pathway takes the major part in the model and insulin signaling pathway is analyzed for its association to learning and memory functions. Validation of the model through serendipitous off-target effects suggests that hormone signaling pathways substantially contribute to the pathogenesis of dementia.

**Conclusions:**

The integrated network model of hormone interactions underlying dementia may serve as an initial translational platform for identifying potential therapeutic targets and candidate biomarkers for dementia-spectrum disorders such as Alzheimer’s disease.

## Background

The clinical hallmark of dementia-spectrum diseases including Alzheimer’s disease (AD) and front temporal dementia is the gradual memory loss and impairment of other cognitive functions, which has been attributed to the aggregation of amyloid fibrils, a process known as amyloidogenesis [1-3]. However, recent findings indicate that many peptide and protein hormones are stored in secretory granules in the form of functional amyloid fibrils and such an amyloid-like structure is necessary for their natural functioning as hormones [4].

Moreover, observational studies on the beneficial effect of estrogen-based hormone therapy on cognitive impairment have also reported conflicting results [5]. Indeed, gender-specific risk profiles observed for dementia in elderly men and women have drawn attention to the impact that sex hormones, as risk factors, may have on progression from mild cognitive impairment (MCI) to dementia [6,7]. The higher risk of AD and dementia incidence in women has been linked to high serum levels of endogenous estrogen [8] and it has been shown to be influenced by hormone replacement therapy [9-13], although a better cognitive performance after current hormone therapy was dependent on the duration and type of treatment [14].

On the other hand, both basic and clinical research findings have consistently shown influence of a range of hormones on some cognitive functions in AD. For example, high levels of leptin in blood have been associated to a lower risk of AD [15] and leptin replacement therapy has been suggested as a novel therapeutic strategy for AD [16]. The loss of melatonin in cerebrospinal fluid has been observed in patients with dementia of Alzheimer’s suggesting that it may play a role in the pathogenesis of AD [17-20]. A low thyroid hormone level has been also associated with AD [21,22]; the administration of thyroid hormone in AD model mice prevented cognitive deficit and improved the neurological function [23]. In Alzheimer’s disease, a greater cognitive impairment has been found to be associated with lower CSF concentrations of corticotropin-releasing hormone [24,25]. There is evidence that growth hormone (GH) declines with advancing age or in Alzheimer’s disease [26-28] and that daily treatment of healthy older adults with GH improves the cognition independent of gender [29]. A recent study also shows that GH can boost memory retention in rats [30].

There are several lines of evidence that point to the role of insulin signaling in AD; e.g. insulin levels in the CSF of AD patients is lower than healthy controls [31,32], insulin receptor signaling is compromised in AD neurons [33], and insulin resistance is associated with reductions in cerebral glucose metabolic rate, which is a risk factor for developing AD dementia [34]. Interestingly, epidemiological findings indicate that type II diabetes mellitus is linked to developing and exacerbating AD pathology [35,36] so that Alzheimer’s has been even proposed by some authors to be ‘type III diabetes’ [37,38]. Similar neuroendocrine disturbances have been reported for Huntington’s disease under which the thyrotropic, somatotropic and gonadotropic axes are altered [39].

All the above-mentioned evidence, including inconsistent results and disparate findings, suggests that there is a gap between the knowledge obtained from basic research and findings of clinical investigations on the association between hormones and cognition. Context-specific networks of molecular interactions provide a relevant framework for supporting translation of basic knowledge into clinically relevant information through integrative modeling of disease mechanism. Current Alzheimer’s disease maps, including the recent Alz Pathway model [40], lack the focused representation of hormone signaling pathways. Therefore, this work describes the first attempt to characterize the hormone/hormone-receptor interactions relevant to dementia disorders under a unified framework of the interconnected hormonal components.

## Methods

**Figure 1:**
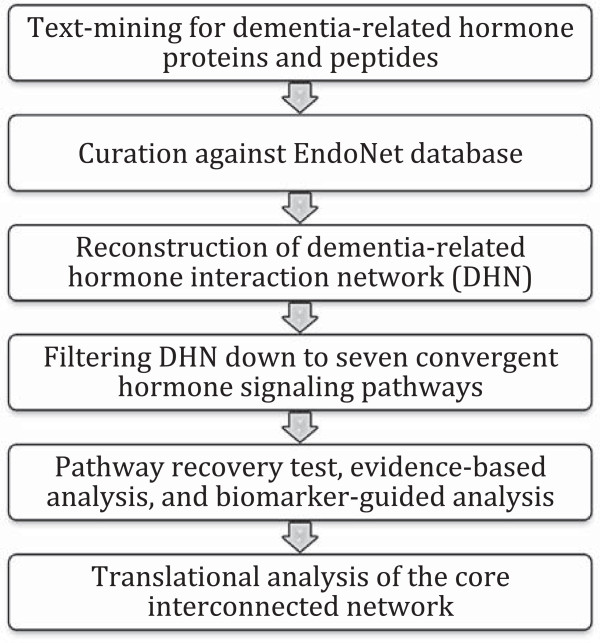
Schematic representation of the methodology used for construction and analysis of dementia-related hormonal network.

Figure 1 summarizes the overall strategy used for this study. It demonstrates a top-down integrative (knowledge- and data-driven) approach to modeling hormone protein interaction network.

### Literature mining

Our retrieval system was composed of two software components: the dictionary-based text-mining tool, ProMiner [41], and the semantic search engine, SCAIView [42]. The ProMiner software uses dictionaries for the recognition of a variety of different terminologies including gene, protein, disease, SNP, drug, etc. The SCAIView software deploys the results from ProMiner annotations, ranks the extracted genes/proteins based on relative entropy scoring function (i.e. mutual information measure), and displays the named entities by tagging them through the text. SCAIView academic version can be freely accessed at http://bishop.scai.fraunhofer.de/scaiview/. PubMed abstracts were searched for all instances of genes and proteins, which are mentioned in the context of ‘dementia’ as keyword (accessed as of 08.02.2011). The retrieved entities were manually checked for their true relevance to both hormones and dementia in the context of their abstracts.

### Network reconstruction and annotation

The results from text-mining were cross-checked with the contents of the EndoNet database [43] and the confirmed entities were used as seed proteins in the BIANA tool for reconstruction of the dementia-related hormonal network (DHN) at level 2 [44]. The initial protein-protein interaction network was constructed around the seed set. In order to reduce the dimensionality and increase the confidence, interactions that are only supported by the yeast two-hybrid method were removed and those interactions that are independently confirmed by two or more experimental methods were maintained. The network was visualized and statistically analyzed in the Cytoscape and Gephi environments [45,46]. G-lay clustering algorithm was used for modularity analysis [47].

### Pathways used for the recovery test

For the pathway recovery test, we obtained the following expert-curated hormonal pathways, used them as gold standard, and mapped them onto the network: growth hormone pathway [48], insulin signaling pathway [48], leptin signaling pathway [49], thyroid hormone signaling [50], regulation of the estrogen receptor pathway [48], corticotropin-releasing hormone pathway [51], and Melatonin signaling pathway [52].

### Statistical analysis

Gene set enrichment analysis was performed using the Molecular Signature Database [53]. DAVID functional annotation tool was used for annotation of differentially expressed genes in the network [54].

### Translational validation

For establishing the clinical relevance of the core DHN model, knockout mouse phenotypes were retrieved from MGI database [55]. For retrieval and extraction of putative biomarker information from the literature, biomarker terminology was used [56]. Pathway membership for each target was obtained from KEGG database [57] and their association to disease was determined using genetic association database [58]. Information on brain tissue specificity of the targets was obtained from Tissue Distribution Database [59] but the higher resolution information at the cell type level was retrieved from the literaure using SCAIView search engine.

DrugBank was searched for the proteins of the core DHN as targets of approved drugs [60]. Then PubMed was searched for supporting evidence of positive off-target effects of non-dementia drugs that showed potential implication of those drug-targets in improvement of dementia.

## Results

### Enrichment of dementia-related proteins for hormone signaling activity

Mining the knowledge space of the literature for proteins that are shown to play a role in dementia resulted in a list of 1960 entities, which were ranked based on their mutual information (see Methods). Due to the fact that high-dimensional information returned by retrieval systems inherits noise, the next step was to observe whether signals of hormonal proteins could be detected in this large list of entities. The gene set enrichment analysis (GSEA) of these proteins revealed under-represented signatures of hormone activities in pathway analysis as well as implicit but statistically significant presence of hormone-related regulatory gene sets in GO biological process annotations (Additional files 1 and 2, respectively). However, at the level of GO molecular function, these signatures showed significant over-representation for hormone activity, neuropeptide hormones and hormone signaling pathways (Table 1).

**Table 1:** Analysis of the retrieved data based on enrichment for gene ontology, category of molecular function

**Gene set name**	**Description of annotation**	**P-value**
Amine receptor activity	GO:0008227: Combining with a biogenic amine to initiate a change in cell activity.	3.61E-05
Hormone activity	GO:0005179: The action characteristic of a hormone, any substance formed in very small amounts in one specialized organ or group of cells and carried (sometimes in the bloodstream) to another organ or group of cells in the same organism, upon which it has a specific regulatory action.	1.46E-04
Copper ion binding	GO:0005507: Interacting selectively with copper (Cu) ions.	3.40E-03
Neuropeptide hormone activity	GO:0005184: The action characteristic of a neuropeptide hormone, any peptide hormone that acts in the central nervous system. A neuropeptide is any of several types of molecules found in brain tissue, composed of short chains of amino acids.	6.55E-03
Serotonin receptor activity	GO:0004993: Combining with the biogenic amine serotonin, a neurotransmitter and hormone found in vertebrates, invertebrates and plants, to initiate a change in cell activity.	1.40E-02

The results of this observation led us to raise the hypothesis that an endocrine interaction network may exist that substantially contributes to the pathology of the dementia-spectrum diseases. To investigate this hypothesis, we used text-mining and knowledge discovery technologies to narrow down our search for retrieval and extraction of instances of hormone proteins and their receptors, which are cited in the literature (Medline abstracts) in relation to dementia. The focused search resulted in retrieval of 1329 documents and 453 protein entities extracted from them. Finally, 89 hormone/hormone-receptor entities were confirmed to play a role as hormone/hormone-receptor after crosschecking the retrieved entities with the contents of the EndoNet database as gold standard (see Methods). We use this initial set of proteins (seed set) as *prior knowledge* to build upon our integrative model.

### Dementia-related hormone network (DHN) and its biological relevance

The initial protein-protein interaction network comprises of 6966 nodes (proteins) and 85997 edges (interactions) but after filtering the number of edges in DHN decreased to 83998. 6515 nodes form a giant connected component and the rest of 451 nodes are singletons without any connection; thus, for simplicity, we only consider the giant component for further analyses. Statistical analysis of the giant component of DHN shows that its node degree distribution could be fitted in the power law of the form y = 1092.8 × ^-1.17^ with an acceptable goodness of fit (R-squared value = 0.856, Correlation = 0.996). This indicates that the network is of biological nature [61].

**Figure 2:**
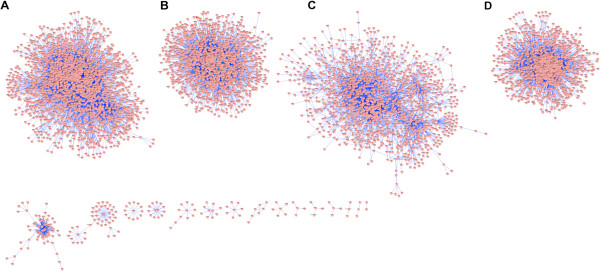
**An overview of modules detected in DHN. **19 modules were detected in DHN out of which 4 modules represent ca. 97% of the network. **(A) **The largest module is enriched for regulation of transcription. **(B) **The second module with 1540 interconnected proteins representing hormone and hormone receptor signaling pathways. **(C) **The third module with 1420 proteins enriched for GPCR signaling. **(D)** The fourth module containing 1312 nodes is enriched for protein translation and induction of apoptosis.

The network clustering coefficient of 0.315 and immense distribution of the clustering coefficients around the nodes with more than 100 neighbours is suggestive of a modular organization consisting of several interconnected functional modules. The modularity analysis of the network revealed four major modules whose functional annotation using GSEA supports the notion of modular organization underlying the network (Figure 2): the largest module with 2037 nodes (Figure 2A) is significantly enriched for regulation of transcription, the second module with 1540 interconnected proteins (Figure 2B) is significantly involved in hormone and receptor signaling, the third module with 1420 proteins (Figure 2C) is significantly annotated for GPCR signaling, and finally the fourth module containing 1312 nodes (Figure 2D) is enriched for protein translation and induction of apoptosis (Additional files 3 and 4). These findings are consistent with the fact that hormone peptides are major ligands for GPCRs and through cellular signaling cascades, they regulate the transcription of target genes in the nucleus.

### Evaluation of DHN by pathway recovery

Both the biological relevance and the modularity were further evaluated by mapping the Alzheimer’s disease pathway from the KEGG database as well as hormone signaling pathways from other resources (see Methods). Mapping the Alzheimer’s disease pathway onto the network resulted in the recovery of all the proteins and their corresponding interactions in the pathway except for APH1A.

Regarding hormone signaling pathways, the number of proteins involved in the actual signaling and the number of mapped proteins for each signaling pathway is shown in Table 2. For two pathways with 100% node recovery, i.e. insulin signaling pathway and growth hormone pathway, manual extraction of edges (interactions) from BioCarta and mapping them onto the network yielded 76% edge recovery (16 out of 21) for the growth hormone pathway and 90% edge recovery (19 out of 21) for the insulin signaling pathway.

**Table 2:** List of dementia-related hormone signaling pathways that were recovered fully or partially in DHN

**Hormone signaling pathway**	**No. of proteins in the gold-standard pathway**	**No. of proteins present in the hormone-dementia network**
Estrogen receptor pathway	30	25 (83% recovery)
Insulin signaling pathway	21	21 (100% recovery)
Growth hormone pathway	27	27 (100% recovery)
Leptin signaling pathway	21	17 (80% recovery)
Thyroid signaling pathway	11	8 (72% recovery)
Melatonin signaling pathway	16	16 (100% recovery)
Corticotropin-releasing hormone signaling pathway	17	16 (94% recovery)

We also surveyed our network for the presence of hormone receptors by comparing them to known hormone receptors of genomic neuroendocrine hormones and were able to identify them for majority of these hormones (Additional file 5).

### Hormonal convergence in DHN

**Figure 3:**
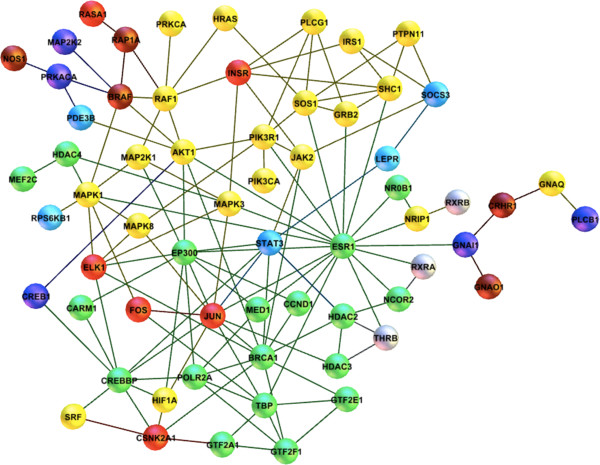
**Elements of the seven hormonal signaling pathways form the core connected component of the brain interactome (normal state with all possible interactions).** Pathway memberships are indicated by color codings; Green: estrogen signaling pathway; Red: insulin signaling pathway; Light blue: leptin signaling pathway; Dark blue: melatonin signaling pathway; Gray: thyroid signaling pathway; Brown: corticotropin-releasing hormone pathway. Yellow color indicates common membership to two or more pathways and also embeds the elements of the growth hormone signaling pathway.

After the completion of this individual pathway recovery test, we aggregated all the elements of these seven pathways and mapped them onto the giant component of DHN. The aim was to detect the core of DHN where the majority of hormone cross talks occur. A subnetwork of 73 nodes and 133 edges was formed, representing the converged hormonal pathway interactions. Interestingly, 62 of these hormone peptides (ca. 86%) are densely interconnected and form the core of DHN. Besides, their interactions appeared to occur in different regions of the normal brain after adding the context of brain region annotations to each edge using the work of Bossi and Lehner (2009) (Figure 3) [62]. Analysis of these annotations shows that the majority of the hormonal interactions occur in prefrontal cortex (ca. 93%), hypothalamus (ca. 92%) and cingulate cortex (ca. 90%), respectively (Additional file 6). The finding that interactions of the converged network mostly occur in prefrontal cortex and cingulate cortex is consistent with the neuroanatomical distribution of neurofibrillary tangles and plaques in the cerebral cortex of AD patients [63]. Moreover, the relevance of this finding to clinical attributes of the advanced AD pathology has been shown in several studies (**Prefrontal cortex:**[64,65]; **Cingulate cortex:**[66]; **Hypothalamus:**[67,68]). For example, it has been shown that prefrontal cortex, an important component for working memory, is the site of hormonal effects on cognition including estrogen [69], insulin [70], growth hormone [71], and thyroid hormone [72]. Thus, collective dysregulation of these pathways in prefrontal cortex of AD patients can lead to worsened memory impairment.

As the pathway-wise color codings in the converged hormonal network in Figure 3 indicates, a strong convergence and close interplay of hormone signals can be observed at the molecular level of the brain interactome. The yellow nodes show the common membership of proteins in two or more of these pathways and are significantly enriched for Neurotrophin/Trk signaling (GSEA p-value: 0e^0^, 14 genes in overlap), through which a variety of signaling cascades are connected and signals of neuronal development, survival as well as additional higher-order signals such as learning and memory are transmitted. The extended portion of estrogen signaling pathway in the core interactome is also noted.

### Linking hormone-dementia hypothesis to mechanistic evidence

Apart from above *in silico* analyses, we provide more solid support for the hormone-dementia hypothesis from Alzheimer’s reference expression data set [73], which has been processed and used for identification of a perturbed protein hub network in Alzheimer’s disease by Liang et al. (2012) [74]. The Alzheimer’s reference data set provides carefully phenotyped expression data set for six brain regions from late-onset AD patients (GSE5281) and lends support to the hypothesis that most of the differentially expressed genes in these six brain regions represent hub proteins in the hub network specific to Alzheimer’s disease. We compared the core DHN with the Alzheimer’s hub network derived from Alzheimer’s reference expression data set and found 18 hormone signaling proteins in the core DHN that overlap with the hub genes differentially expressed in the hub network of Alzheimer’s disease (Additional file 7). As Additional file 7 indicates, all hormone signaling pathways are perturbed in different brain regions, with the largest overlap between insulin and growth hormone signaling pathways.Among these proteins, ESR1 and IRS1 exclusively represent two hormone signaling pathways, namely estrogen signaling pathway and insulin signaling pathway.

### Translational validation of the core DHN

To our knowledge, except for hormone therapy with estrogen, there is no clinical trial describing the effect of other hormones on cognition improvement. Hence, in the absence of clinical trials, we propose a strategy for translational validation of the core DHN by showing the clinical relevance of the core DHN to dementia in the first step and then linking molecular signatures - through mouse model phenotypes - to their corresponding clinical manifestations.

The clinical relevance of the core DHN to dementia can be established through biomarker-guided analysis, in which information of putative molecular indicators of dementia is retrieved and extracted from the literature and further become enriched with pathway membership, disease association and tissue/cell type specificity data (Table 3). Of the proteins in the core DHN, four were found in the literature to be reported as potential biomarkers that show measurable activity under Alzheimer’s condition. These four proteins represent four different hormonal signaling pathways, namely growth hormone pathway (MAPK3), corticotropin-releasing hormone pathway (NOS1), melatonin signaling pathway (CREB1)and insulin pathway (JUN), whose measurable activities under AD condition suggest their mechanistic involvement in the pathology of AD dementia.

**Table 3:** Clinical relevance of the core DHN to dementia through biomarker-guided analysis

**Target candidate**	**Pathway membership**	**Disease association**	**Biomarker type**	**Brain tissue specificity**	**Cell-type specificity**
MAPK3	Alzheimer’s disease, Prion disease, Type II diabetes mellitus, Insulin signaling pathway, Long-term potentiation	Autism	CSF increased levels AD (PMID: 22145083, 19625747), Phosphorylation (PMID: 19233276, 16920298, 17612901), Alterations in lymphoblasts of AD patients (PMID: 19158936)	Left ventricle, Right ventricle, Brain stem	Microglia, Astrocytes, Neurons
NOS1	Alzheimer's disease, Long-term depression, Calcium signaling pathway	Parkinson's disease, Alzheimer's disease, Diabetes Mellitus type II	Nitric oxide overproduction (PMID: 20804853), nNOS signaling initiated in interneurons (PMID: 16758165), Increased expression of nNOS isoforms in astrocytes (PMID: 12384247)	Substantia nigra, Forebrain, cerebral white matter, Limbic system	Astrocytes, Neurons
CREB1	Huntington's disease, Cholinergic synapse	Alzheimer's disease	Impaired CREB phosphorylation (PMID:22119240)	Substantia nigra, Brain stem, Sub-commissural organ, Brain ventricle, Cerebral gray matter, Cerebral white matter, Forebrain, limbic system	Hippocampal neurons, Dendate gyrus
JUN	GnRH signaling pathway, Neurotrophin signaling pathway, MAPK signaling pathway	Cognitive performance	Prolonged expression of c-Jun (PMID:8774439), Increased immuno-reactivity (PMID:8313943)	Sub-commissural organ, Brain ventricle, Cerebral gray and white matter	Neurons, Microglia, Substantia nigra

Next, we sought to investigate the translational value of DHN by linking hormone proteins in DHN to their corresponding knockout mouse phenotypes. Table 4 summarizes 19 knockout mouse models representing 6 hormonal signaling pathways with phenotypes related to the nervous system. It also includes the ratio of knockout studies reporting an effect on the nervous system to studies reporting no effect on the nervous system.

**Table 4:** Knockout mouse phenotypes observed for several proteins in the core DHN model

**Name**	**Mutation category**	**Observed effects on the nervous system**	**Ratio of KO studies with CNS phenotypes to studies without CNS phenotypes**
**Estrogen signaling**			
Esr1^tm1Ksk^	Targeted (knock-out)	abnormal pituitary gland physiology	4:8 (50%)
		abnormal hypothalamus morphology	
		abnormal innervation	
Ncor2^tm1Kjep^	Targeted (knock-out)	abnormal cerebral cortex morphology	1:0 (100%)
		abnormal neuron differentiation	
Hdac2^tm1.2Rdp^	Targeted (knock-out)	abnormal hippocampus CA1 region morphology	1:3 (34%)
		abnormal dentate gyrus morphology	
		abnormal hippocampus pyramidal cell morphology	
		enhanced long term potentiation	
Ccnd1^tm1Wbg^	Targeted (knock-out)	absent Purkinje cell layer	1:3 (34%)
		abnormal cerebellar granule layer	
		small cerebellum	
Crebbp^tm1Sis^	Targeted (knock-out)	abnormal forebrain morphology	3:4 (75%)
**Insulin signaling**			
Jun^tm1Wag^	Targeted (knock-out)	abnormal forebrain morphology	1:2 (50%)
Hras1^tm1Grnt^	Targeted (knock-out)	reduced long term potentiation	1:3 (34%)
Csnk2a1	Targeted (knock-out)	abnormal telencephalon development	1:2 (50%)
Mapk3^tm1Gpg^	Targeted (knock-out)	reduced long term potentiation	2:2 (100%)
**Leptin signaling pathway**			
Lepr^tm1.2Chua^	Targeted (knock-out)	abnormal inhibitory postsynaptic currents	1:4 (25%)
Stat3^tm1Aki^	Targeted (knock-out)	abnormal motor neuron morphology	1:6 (17%)
		abnormal neuron physiology	
Hif1a^tm1.1Stom^	Targeted (knock-out)	abnormal cerebrum morphology	4:7 (57%)
		abnormal cerebral cortex morphology	
		loss of cortex neurons	
		abnormal occipital lobe morphology	
		abnormal temporal lobe morphology	
		loss of hippocampal neurons	
**Thyroid signaling pathway**			
Rxrb^tm1Rev^	Targeted (knock-out)	abnormal excitatory postsynaptic potential	2:3 (67%)
		reduced long term potentiation	
		absent long term depression	
**Corticotropin-releasing pathway**			
Gnaq^tm1Soff^	Targeted (knock-out)	abnormal glutamate-mediated receptor currents	1:2 (50%)
		absent long term depression	
Braf^tm1.1Sva^	Targeted (knock-out)	increased neuron apoptosis	3:3 (100%)
		abnormal innervation	
		thin cerebral cortex	
Nos1^tm1Plh^	Targeted (knock-out)	abnormal brain wave pattern	3:4 (75%)
		abnormal long term potentiation	
		reduced long term potentiation	
		absent long term depression	
		decreased synaptic glutamate release	
		abnormal peripheral nervous system regeneration	
**Melatonin signaling pathway**			
Gnai1^tm1Drs^	Targeted (knock-out)	abnormal long term potentiation	1:1 (100%)
Plcb1^tm1Hssh^	Targeted (knock-out)	loss of hippocampal neurons	1:1 (100%)
Creb1^tm1Gsc^	Targeted (knock-out)	abnormal CNS synaptic transmission	1:2 (50%)
		reduced long term potentiation	

To establish the bridge between the observed mouse phenotypes and the clinical disease manifestation in human, we propose the novel concept of “mechanism discovery through serendipitous off-target effects” based on the secondary positive effect of approved drugs that leads to unexpected and serendipitous clinical observations. Many approved drugs that are routinely used for treatment of human diseases lead to manifestation of so-called ‘hidden phenotypes’ due to binding to unknown targets [75]. The revelation of hidden phenotypes points to the fact that off-target effects sometimes result in positive effects through novel mechanisms of action. The most prominent example is the positive effect of Sildenafil on erectile dysfunction while the drug had been originally developed against angina.

**Figure 4:**
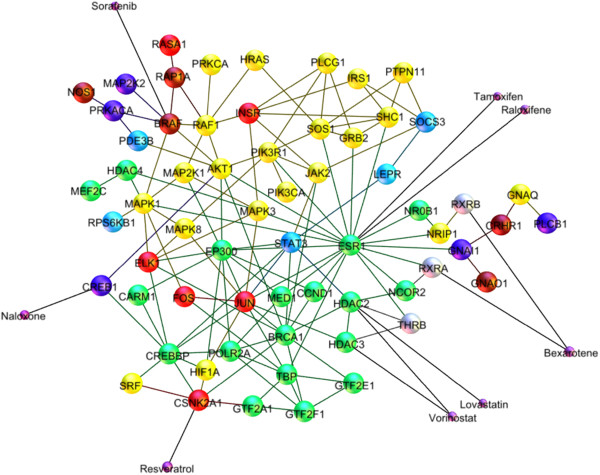
Schematic off-target landscape of 8 non-dementia drugs in the core DHN model.

Here we have collected a number of drugs with reported serendipitous effects on cognition that target several proteins in the core DHN (Table 5). Interestingly, all the off-targets of these drugs, when compared to the knockouts in Table 4, correspond to a nervous system phenotype in mice. Landscape illustration of the off-target effects in the core DHN model in Figure 4 shows that modulation of the estrogen signaling pathway by four drugs was more likely to lead to the serendipitous off-target effect on dementia and consequently, to improved cognitive functions observed in patients treated with these drugs.

**Table 5:** Drugs with serendipitous off-target effects on cognition and memory

**Drug name**	**Main indication**	**Positive side effect on cognition and memory**	**Study subjects/design**	**Supporting evidence (PMID)**	**Target protein in core DHN**
Bexarotene (Targretin)	Skin cancer	Rapid reversal of cognition, social and olfactory deficits	Mouse model of AD	22323736	RXRA
					RXRB
Tamoxifen	Breast cancer	Higher level of independence in activities of daily life and decision making; relationship of tamoxifen with a lower prevalence of AD	Cross-sectional study of women receiving tamoxifen	11005221	ESR1
Raloxifene	Breast cancer	Reduced risk of cognitive impairment in postmenopausal women	The Multiple Outcomes of Raloxifene Evaluation (MORE) randomized, placebo-controlled trial amonf postmenopausal women with osteoporosis	15800139	ESR1
Vorinostat	Cutaneous T cell lymphoma (skin cancer)	Complete restoration of contextual memory	Mutant APPswe/PS1dE9 mice	20010553	HDAC2
					HDAC3

Lovastatin	Hyperlipidemia	Reduction of Abeta formation and slowing the progression of AD	Double-blind randomized study on human subjects	11900994	HDAC2
Resveratrol	Aging	Promoting clearance of Abeta peptides	Various cell lines	16162502	CSNK2A1
Sorafenib	Renal cell carcinoma	Reversal of memory impairment	Transgenic APPswe mouse model	20201822	BRAF
Naloxone	Opioid overdose	Improvement of learning and memory through enhancement of long-term potentiation	Aged rats with declined memory	14670637	CREB1
				15805661	

To enlighten the usability of the DHN model, we performed a more general analysis by systematically searching for non-dementia drugs with targets of the core DHN model and retrieving published studies that support the positive, negative or neutral effect of those drugs on cognition or memory or learning. This analysis demonstrated that of 62 proteins in the core DHN model, 21 (ca. 33%) have been already targeted by at least one drug out of which 18 drugs targeting 13 proteins have shown positive effect, 3 drugs targeting 1 protein have demonstrated negative effect, and 39 drugs targeting 18 proteins have not been investigated in relation to cognitive functions or have not been reported in the literature to have any observed effect on dementia. 21 proteins (ca. 33%) have been targeted by experimental compounds and 20 proteins (ca. 32%) have not been targeted by any drug or compound (Additional file 8). These findings imply that hormone signaling pathways present a promising target space for drug discovery.

## Discussion

Integrative modeling approaches provide a suitable medium for fusion of complementary data derived from literature and experiments. Given the fact that molecular mechanism of disease risk factors is often unclear and the exact mode of action of most approved drugs is unknown in most cases, such models can be used to interpret disease mechanism and to predict drug mode of action. In particular, an integrative model allows for inference of cross talks among components of the system, guidance of analysis to the core pathological pathways, and generation of further hypotheses on how risk factors or disease-modifying treatments at molecular level lead to manifestation of positive or negative clinical effects. Accordingly, our in silico approach to modeling hormone signaling pathways that underlie dementia pathology provided several novel insights beyond what is already known about hormone signaling pathways in dementia, as follows.

The growing number of findings on the role of hormone signaling pathways in regulation of cognition and memory raises an immediate question: how these bits and pieces of accumulating knowledge are being used to explain the contribution of hormones to improvement or exacerbation of dementia? The dementia-related hormonal network, presented in this paper, provided a first unified picture of the hormonal component underlying cognitive impairment. Convergence of genomic hormonal pathways in the DHN model uncovered tight molecular interconnections and cross talks among hormone signaling pathways and regulatory pathways of neural growth, survival and differentiation. For instance, the observed convergence of estrogen and neurotrophin signaling pathways at the core of DHN has been shown to regulate an array of cytoskeletal and growth-associated genes in cerebral cortex, including tau microtubule associated protein, MAPT [76]. The implication of such hormone signals in the pathology of dementia is supported by the evidence that phosphorylation of MAPT, which leads to neurofibrillary tangle formation and ultimately neurodegeneration, is regulated by the signaling effects of insulin and estradiol [77,78]. Similarly, the regulatory influence of thyroid hormone, melatonin, and corticotropin-raleasing factors on hippocampal tau phosphorylation has been documented in the literature [79-81].

The DHN model could guide the mechanism discovery analysis to those signaling pathways that constitute the core pathological processes. The modularity detected in the network implies that hormone receptors and hormone signals in concert with transcription factors may play a significant part in the disease mechanism. The molecular interconnection of insulin pathway to dementia pathology - revealed by the DHN model– may provide a mechanistic explanation for the previous epidemiological studies on the contribution of diabetes mellitus and insulin resistance as risk factors to exacerbation of dementia e.g. [82-84]. For instance, very recently, a 9-year prospective study on 3069 elderly adults without dementia demonstrated that patients who suffered diabetes had significantly worse cognitive decline in comparison with those who did not have the disease, suggesting the contribution of diabetes mellitus severity to accelerated cognitive impairment [85]. An interesting observation in our model is the co-occurrence of diabetes-related proteins in the convergent core of DHN. Indeed, three members of this subnetwork (i.e. MAPK1, INSR, SOCS3) belong to the Type II diabetes mellitus pathway, which fall into the bigger insulin signaling pathway together with SHC1 and ELK1 (Figure 3). As was shown by pathway recovery analysis in Table 2, the insulin signaling pathway is present in the network with the highest number of nodes and edges amongst other signaling pathways. The presence of MAPK1/ELK1/CREBBP axis in the core subnetwork (see Figure 3) and its direct crosstalk to the insulin pathway is consistent with experimental observations that link insulin signaling and diabetes risk to the regulation of learning and formation of long-term memory [86-88].

We showed that the DHN model could have more valuable implications beyond a sole portrait of networked signaling pathways by enabling high-resolution analysis of core molecular events. This was achieved through enhancement of the DHN model with knockout phenotype data and drug-target information. Genetically engineered mouse models play an instrumental role in studying disease mechanism and translating preclinical studies to the clinic [89]. Thus, the knockout phenotypes are good candidates for establishing the link between the molecular mechanism and the disease clinical manifestation. One clear observation from knockout phenotypes in Table 4 is the prominent involvement of all hormone signaling pathways in long term potentiation (LTP) beside other biological processes. It is well known that long term potentiation of synaptic transmission substantially contributes to memory formation [90] and that LTP inhibitors also block memory and learning [91]. Hence, it can be inferred from the model that probably pertubarion in hormone signaling pathways may affect LTP adversely. Interestingly, knockout models of three putative biomarkers for dementia in Table 3, namely MAPK3, NOS1 and CREB1, show reduced LTP (see Table 4), which supports the notion that hormone signaling pathways are part of the dementia pathology. It should be noted that these proteins generally exert multiple functions in the biology of nervous system by participating in different signaling pathways and thus, the core DHN model describes their contribution to the hormone-mediated signaling in the context of dementia.

Since mouse knockout phenotypes alone might not be sufficient to concretely conclude about the translational value of our DHN model, introduction of the “serendipitous off-target effect” for linking DHN model to disease mechanism demonstrated to provide further validation for the DHN model. It was shown that inhibition of off-targets belonging to hormone signaling pathways could lead to improvement of memory and learning in human or animal models. Therefore, the enhanced DHN model can be used to predict novel targets out of off-targtes or to identify disease-modifying targets and pathways that partially regulate the pathology of disease.For example, HDAC2 knockout mouse models show enhanced LTP, which may indicate HDAC2 might be a potential therapeutic target; on the other hand, the clinical evidence is provided by the off-target effect of Lovastatin on HDAC2: originally designed against hypercholesterolemia, Lovastatin was tested during a double-blind, randomized clinical study on human subjects for its effect on progression of Alzheimer’s disease through reduction of amyloid-beta formation [92]. The study found that Lovastatin decreases the risk of AD progression. Such an inference exemplifies how the novel knowledge on the mechanism of drug effect on disease-related risk factors can be derived from the enhanced integrative model of DHN.

Although DHN provides a unified integrative map of possible hormone signaling mechanism in the context of dementia, it has its limitations. The inherent issue of network biology is that completeness of molecular network maps is limited to data availability and validity. The DHN model analyzed in this work may not cover all the hormone pathways involved in the pathogenesis of dementia but rather it focuses on the convergent hormone action by the most prominent ones. Furthermore, such models provide only a static picture and do not capture the dynamic behaviour of the system. However, context-specific modeling, as the first step, makes it possible to simulate disease-specific perturbations after incorporation of quantitative data from high-throughput technologies. Such an integrative modeling approach may prove valuable for prediction of potential biomarkers due to the fact that hormones are able to cross the blood–brain barrier by transmembrane diffusion or using transporters and their brain levels reflect blood levels [93]. We plan to keep the DHN model up to date –within the boundaries of available resources –by implementing an alert system that automatically collects new information published on the role of hormone signaling in dementia and enriches the model with the emerging knowledge. It is anticipated that, with the availability of more data, the resolution (i.e. specificity and sensitivity) of the model will increase so that new versions of the model will support translational scientists to make informed decisions.

## Conclusions

The integrated hormone interaction model presented in this study can be beneficial in correlating the information of genes, proteins, signaling pathways and the clinical manifestation of dementia in the context of endocrine system. Such models have great potential to support the process of identifying new targets and novel biomarkers and help the pharmaceutical industry to increase the efficiency of their pipeline.

## Competing interests

Authors declare no competing interests.

## Authors’ contributions

EY conceived of the study, designed the methodology, performed analyses, and drafted the manuscript. MHA revised the results critically and supported the study. All authors read and approved the final manuscript.

## Supplementary Material

Additional file 1 **Analysis of dementia-related proteins for pathway enrichment. **Results of GSEA pathway analysis on the list of dementia-related genes/proteins that were retrieved from the literature show a weak indication for presence of a subtle hormone signaling component. Description: Result table of GSEA pathway analysis on the list of literature-derived genes/proteins. Click here for file

Additional file 2 **Analysis of dementia-related proteins for GO biological processes enrichment.** Results of GSEA GO (Biological Process) analysis again point to subtle hormone-dependent biological processes. Description: Result table of GSEA GO biological process analysis on the list of literature-derived genes/proteins. Click here for file

Additional file 3 **Module subnetworks of DHN. **Module subnetworks - produced by cluster analysis of DHN – have been provided in a single XGMML file, which can be loaded, viewed and navigated through the Cytoscape software. Description: Subnetworks of DHN representing modules enriched for edge attributes. Click here for file

Additional file 4 **Enrichment analysis results for each module subnetwork of DHN. **Detailed results of gene set enrichment analysis for proteins of four giant components (modules) using the category of GO Biological Process. Description: Result table of GSEA GO biological process analysis on each DHN modules. Click here for file

Additional file 5 **List of genomic neuroendocrine hormones and their corresponding receptors in DHN.** The table lists the genomic hormones, their coding genes, their presence in the DHN model, and their corresponding receptor proteins in DHN. Description: Genomic hormones and their corresponding receptors have been retrieved from literature and manually curated. Click here for file

Additional file 6 **Annotations of DHN interactions to brain regions. **Protein-protein interactions in DHN have been annotated to brain regions where they interact (1 represents an interaction and 0 represents no interaction). Description: Annotation of DHN protein interactions to brain regions supported by extra information. Click here for file

Additional file 7 Putative AD biomarkers in the core DHN supported by gene expression data and pathway membership. Click here for file

Additional file 8 **General analysis of the drug-target space in the core DHN model. **Proteins in the core DHN model have been annotated with all non-dementia drugs that target them and supporting evidence from the literature reporting positive (highlighted in green), negative (highlighted in red), and neutral (highlighted in grey) effects on cognition or memory or learning. Description: Systematic analysis of the drug-target space in the core DHN model for off-target effects on dementia. Click here for file
