# Supplementary material for: A network model of genomic hormone interactions underlying dementia and its translational validation through serendipitous off-target effect
Source: J Transl Med. 2013 Jul 26;11:177. doi: 10.1186/1479-5876-11-177 (PMC3733613; doi:10.1186/1479-5876-11-177)
Supplement: Additional file 5 — List of genomic neuroendocrine hormones and their corresponding receptors in DHN. The table lists the genomic hormones, their coding genes, their presence in the DHN model, and their corresponding receptor proteins in DHN. Description: Genomic hormones and their corresponding receptors have been retrieved from literature and manually curated. [file 1479-5876-11-177-S5.doc]

Additional file 5 – List of genomic neuroendocrine hormones and their corresponding receptors in DHN

| **Hormone** | **Gene** | **Presence in hormone-dementia network** | **Corresponding hormone receptor(s) in the network** |
| --- | --- | --- | --- |
| Adrenocorticotropin (ACTH) | POMC | Yes | MC2R |
| Arginine vasopressin (AVP) | AVP | Yes | AVPR1A, AVPR1B, AVPR2, VCAM1 |
| Corticotropin-realising hormone (CRH) | CRH | Yes | CRHR1, CRHR2 |
| Endorphin | POMC | Yes | OPRL1, OPRM1, OPRK1, OPRD1 |
| Enkephalin | PENK | No | OPRL1, OPRM1, OPRK1, OPRD1 |
| Growth hormone (GH) | GH1 | Yes | GHR |
| Luteinizing hormone-releasing hormone (LHRH) | GNRH1 | Yes | GNRHR |
| Luteinizing hormone (LH) | LHB | Yes | LHCGR |
| Neurophysin | OXT | Yes | OXTR |
| Prolactin | PRL | Yes | PRLR |
| Somatostatin | SST | Yes | SSTR1, SSTR2, SSTR3, SSTR4, SSTR5 |
| Substance P | TAC1 | Yes | TACR1, TACR2, TACR3 |
| Thyroid-stimulating hormone (TSH) | CGA | Yes | TSHR |
| Vasoactive intestinal peptide (VIP) | VIP | Yes | VIPR1, VIPR2, ADCYAP1R1 |
| Growth hormone-releasing hormone | GHRH | Yes | GHRHR |
| Prolactin releasing hormone | PRLH | No | PRLHR |
| Thyrotropin-releasing hormone | TRH | Yes | TRHR |
| Follicle-stimulating hormone | FSHB | Yes | FSHR |
