# Supplementary material for: A network model of genomic hormone interactions underlying dementia and its translational validation through serendipitous off-target effect
Source: J Transl Med. 2013 Jul 26;11:177. doi: 10.1186/1479-5876-11-177 (PMC3733613; doi:10.1186/1479-5876-11-177)
Supplement: Additional file 7 — Putative AD biomarkers in the core DHN supported by gene expression data and pathway membership. [file 1479-5876-11-177-S7.doc]

**Additional file 7: Table S3. Putative AD biomarkers in the core DHN supported by gene expression data and pathway membership.**

|  | **Visual Cortex** | **Superior Frontal Gyrus** | **Posterior Cingulate Cortex** | **Middle Temporal Gyrus** | **Hippocampus** | **Entorhinal Cortex** | **Estrogen Receptor Pathway** | **Insulin Signaling Pathway** | **Growth Hormone Pathway** | **Leptin Pathway** | **Thyroid Pathway** | **Melatonin Pathway** | **Corticotropin Pathway** |
| --- | --- | --- | --- | --- | --- | --- | --- | --- | --- | --- | --- | --- | --- |
| AKT1 |  |  |  |  |  |  |  |  |  |  |  |  |  |
| BRCA1 |  |  |  |  |  |  |  |  |  |  |  |  |  |
| CREBBP |  |  |  |  |  |  |  |  |  |  |  |  |  |
| CSNK2A1 |  |  |  |  |  |  |  |  |  |  |  |  |  |
| EP300 |  |  |  |  |  |  |  |  |  |  |  |  |  |
| ESR1 |  |  |  |  |  |  |  |  |  |  |  |  |  |
| IRS1 |  |  |  |  |  |  |  |  |  |  |  |  |  |
| JUN |  |  |  |  |  |  |  |  |  |  |  |  |  |
| MAP2K1 |  |  |  |  |  |  |  |  |  |  |  |  |  |
| MAPK1 |  |  |  |  |  |  |  |  |  |  |  |  |  |
| MAPK3 |  |  |  |  |  |  |  |  |  |  |  |  |  |
| PIK3R1 |  |  |  |  |  |  |  |  |  |  |  |  |  |
| PLCG1 |  |  |  |  |  |  |  |  |  |  |  |  |  |
| PRKCA |  |  |  |  |  |  |  |  |  |  |  |  |  |
| PTPN11 |  |  |  |  |  |  |  |  |  |  |  |  |  |
| RAF1 |  |  |  |  |  |  |  |  |  |  |  |  |  |
| SHC1 |  |  |  |  |  |  |  |  |  |  |  |  |  |
| STAT3 |  |  |  |  |  |  |  |  |  |  |  |  |  |
